# Nursing students' experience during their practicum in an intensive care unit: A qualitative meta-synthesis

**DOI:** 10.3389/fpubh.2022.974244

**Published:** 2022-09-29

**Authors:** Yue Liu, Lingmin Wang, Haiyan Shao, Peng Han, Jinxia Jiang, Xia Duan

**Affiliations:** ^1^Emergency Department, Shanghai Tenth People's Hospital, School of Medicine, Tongji University, Shanghai, China; ^2^Nursing Department, Shanghai First Maternity and Infant Hospital, School of Medicine, Tongji University, Shanghai, China

**Keywords:** experience, intensive care unit, meta-synthesis, nursing students, preceptorship, qualitative systematic review

## Abstract

**Background:**

Clinical practicum provides nursing students with more opportunities to learn their professional knowledge and develop basic nursing skills. Intensive care unit (ICU) is often used as one of the clinical practicum departments for nursing students. Due to the characteristic fast-paced working environments, high acuity of patient care, and technical complexities of an ICU, nursing students are more susceptible to experiencing stress and lack of confidence in these settings, which hinders their professionalization and affects patient care.

**Objective:**

The study aimed to summarize and evaluate the nursing students' experience in an ICU during their practicum and to provide a supportive ICU clinical practicum environment for them. One of the main objectives was to increase the ICU specialty nurse reserve and improve nursing care in the ICU.

**Methods:**

The following databases were searched for related qualitative publications in Chinese and English by systematic searches across January 2022, including the nursing students' experience in ICU during their practicum: PubMed, Cochrane Library, Web of Science, and so on. The qualitative meta-synthesis was conducted according to the Preferred Reporting Items for Systematic Reviews and Meta-Analyses (PRISMA) recommendations. Two reviewers independently selected these studies and carefully evaluated the quality of each study. Meta-synthesis was then used to summarize the results.

**Results:**

Eleven sub-themes and 3 themes were revealed in 9 studies: challenges of clinical practicum in the ICU, the expectation of support from multiple sources, and the importance and necessity of practicum in the ICU.

**Conclusion:**

Performing one's practicum in ICU was considered by the nursing students in this review as a beneficial practicum despite the challenges involved. The appropriate guidance and monitoring should be given by hospital managers and college educators.

## Introduction

Nursing is a practice-based discipline, and the nursing education program consists of college theoretical education and clinical practicum education (1). The purpose of college theoretical education is to enable nursing students to obtain basic theoretical knowledge and clinical technology. Also, the ultimate purpose of clinical practicum education is to help integrate theoretical knowledge into clinical practice of nursing students and cultivate their clinical competencies (2).

Clinical practicum education is a necessary stage to complete the transition of the nurse roles for nursing students (3) and provides nursing students the environment to master all the knowledge, emotional and professional technology that they have learned during their college theoretical education and are expected to acquire at graduation (4). According to the literature, significant differences exist in the arrangement of clinical practicum education in nursing institutions worldwide. For example, in a 4-year-long nursing undergraduate education, students are required to complete a certain number of clinical practicum hours as part of their credits. These hours vary from 2,800 h in South Africa, 2,300 h in the United Kingdom, 1,400 h in Hong Kong, 1,100–1,500 h in New Zealand, to 800 h in Australia (5–7). To help nursing students prepare and transition to a clinical practicum environment, many medical schools have implemented measures such as short transition courses or clinical clerkship (8). Clinical practicum education in Iran starts in the 1st year together with college theoretical education. The theoretical education is completed by the end of the third academic year, and students work independently in clinical wards under the supervision of clinical instructors in the fourth academic year (9). In mainland China, nursing students need to accomplish 40 weeks of full-time clinical practicum in their senior year (10). Nursing students in Turkey study clinical applications for 3 weeks in their 2nd and 3rd years. Final-year nursing students serve as interns 4 days a week for clinical practicum (4, 11). On the one hand, nursing students can apply their professional knowledge and skills in a real environment and observe the current system operations during their clinical practicum. On the other hand, they can also learn how to assess and manage patients. Moreover, they have the opportunity to develop a professional identity, critical thinking, communication skills, and empathy and increase self-confidence by taking responsibility for patient care (12). Without a doubt, clinical practicum education is a crucial component of nursing education (13).

Intensive care unit (ICU) practicum plays an important role in clinical practicum education, usually scheduled for 160–280 h, accounting for nearly 10% of the total practicum time (4, 14). The ICU, also known as an intensive therapy unit or intensive treatment unit or critical care unit (CCU), is a unit that provides advanced life-support and treatment for critically ill patients with complex conditions (15). According to the type of patients treated, the ICU is divided into emergency intensive care unit, respiratory intensive care unit, neurosurgical intensive care unit, and so on. Because most of the patients in the ICU have chronic underlying diseases with complex conditions, low immune functions, and disordered pathophysiological conditions, a qualified ICU nurse should possess profound knowledge, critical thinking, rapid decision-making, and ability of multidisciplinary cooperation besides the clinical competencies of ordinary nurses. Owing to the aforementioned characteristics, the ICU has been recognized as providing students with the opportunity to acquire a variety of technical skills (e.g., continuous renal replacement therapy, invasive or non-invasive mechanical ventilation, endotracheal tube suction, and various life-support and rehabilitation technologies) and to learn through an interdisciplinary approach and develop communication skills with critically ill patients, which cannot be available in other wards (16). At the end of the practicum in the ICU, nursing students are required to be familiar with professional knowledge, observation, nursing, and rescue of critically ill patients, as well as various commonly used monitoring technologies, and to become critical thinkers, collaborators, and team players ultimately (17). The training requirements for nursing students in the ICU are higher than those in other wards.

As discussed previously, extensive research has shown that ICU provides an excellent learning opportunity for students but inevitably brings some stress (4, 11, 18). The sources of the stress of nursing students in an ICU include the complex environment, advanced technological equipment used, relationships with the clinical instructors and peers, characteristics of the critically ill patients themselves, and unique challenges of caring for patients with an unpredictable public health emergency, such as coronavirus disease 2019 (COVID-19) (11). COVID-19 can cause a wide variety of symptoms ranging from asymptomatic infection to severe complications such as acute respiratory distress, metabolic acidosis, multiple organic failure, and eventually even death (19). On average, 25% of COVID-19 patients received treatment in ICUs (range 5–32% depending on the institution and country) (20, 21). Intensive care for patients with COVID-19 typically includes routine oxygen therapy, non-invasive and invasive ventilation, routine procedures of acute respiratory distress syndrome and mechanical circulatory support (e.g., extracorporeal membrane oxygenation) (20). Without a doubt, this puts forward higher requirements on nursing students' learning. Meanwhile, the demand for critical care in many countries exceeds the capacity of hospitals. For this reason, the COVID-19 pandemic has presented a precedented challenge not only for nursing students but also for intensive care systems across the globe (22). Therefore, cultivating the knowledge and skills required by nursing students to care for and manage critically ill patients is highly important to increase the ICU specialty nurse reserve.

According to a previous study, nursing students' experience reflected that practicum in the ICU provided various benefits for them (18). However, whether the current clinical practicum in the ICU is suitable for nursing education because of its complicated nature and overstimulation is not clear (23). Define nursing students' experience in ICU for evaluating clinical practicum is essential. Meanwhile, the education systems in various countries and regions are attaching great importance to the protection of nursing students, but limited (4, 11). Therefore, the purpose of the present study is to synthesize the literature on the practicum experience of nursing students in the ICU and to further provide a supportive ICU clinical learning environment for them. One of the main objectives was to increase the ICU specialty nurse reserve and improve nursing care in ICUs.

## Methods

### Design

The purpose of this review is to identify, evaluate, and integrate data from qualitative researches that describe nursing students' experience during their practicum in an ICU. Qualitative synthesis follows the recommendations of the Preferred Reporting Items for Systematic Reviews and Meta-Analysis (PRISMA) (24). Detailed information is presented in Appendix Figure 1 for PRISMA flowchart. Searching for relevant articles, extraction of data, and making critical assessments using thematic synthesis were based on the three steps outlined by Thomas and Harden (25): (1) coding the text line-by-line, (2) organizing codes descriptive themes, (3) developing analytical themes.

### Search methods

Qualitative studies published in Cochrane Library, PubMed, Embase, Ovid, Elsevier, Web of Science and Chinese database, including China Biomedical Database (CBM), Wanfang Database (CECDB), Chinese National Knowledge Infrastructure (CNKI) and China Science and Technology Journal Database (VIP Database), were conducted the search by two researchers in January 2022.

The researchers searched using terms and subject headings, and adjusting for different databases. Boolean operators were used to combine keywords or MeSH terms: (1) nursing student^*^, internship^*^, and student^*^ in nursing; (2) ICU^*^, intensive care unit, and critical unit; (3) preceptorship, clinical practicum, clinical practice, and clinical education; and (4) qualitative research^*^, feelings, experience and perceptive. One author reviewed all titles and abstracts for eligibility of the potentially relevant studies.

### Inclusion and exclusion criteria

#### Study design

The qualitative research from which qualitative data could be extracted was selected. The main qualitative research methods include but are not limited to phenomenology, grounded theory, action research, and ethnography and so on.

#### Participant

Nursing students in the ICU during their practicum were included in the study.

#### Interest of phenomena

Nursing students' experience during their practicum in the ICU was considered for the study.

#### Context

Nursing students who had completed or were continuing to practicum program in the ICU were included in the study.

#### Exclusion criteria

Researches that were non-qualitative or collected qualitative data but used quantitative methods for analysis were excluded. Studies not published in English or Chinese, studies not published in peer-reviewed journals, case reports, conference proceedings, poster abstracts, and so on were excluded. Systematic reviews were not included, but their references were reviewed to determine possible relevant studies.

### Search outcomes

Two authors independently selected literature and extracted information based on the inclusion and exclusion criteria. Three hundred seventy-six articles were yielded in the initial search using the aforementioned strategies. First, the researchers read the titles and abstracts of the articles, and excluded articles that were unrelated to the topic, repetitive, and not available in full. The next 351 articles were not included. After reading the whole text, 17 articles were not included. Eventually, nine related articles were included in the study.

### Quality appraisal

The two first authors independently assessed the methodological quality of the nine included articles. Initially, the Joanna Briggs Critical Assessment Tool for Methodological Quality Assessment was used by the authors (26). This consists of 10 questions that are used to quickly and effectively assess the studies, each answered with a “yes,” “no” or “unclear.” A score was assigned to each criterion (yes = 2, no = 0, unclear = 1), giving the maximum score of 20 for each study. Then these scores were converted into percentages. If any differences arise, disagreements were resolved through discussion. Because all studies scored above 70%, none of them were excluded from the quality assessment process (Appendix Table 1).

### Data extraction

The researchers conducted a comprehensive study to describe the quality of the content and to evaluate the methodological development in the included studies (27, 28). The information was extracted on the authors, publication year, country and region, research methodology, participants, aims, and main results. All the results were cross-examined by two researchers, and any differences were addressed through discussion with the third researcher. The summary results are shown in Appendix Table 2.

### Data synthesis

Meta-aggregation was used to integrate the results of the qualitative researches (26). First, every included research was reread several times to increase familiarity and gain insight into research objectives, methodology, and findings. Every discovery was then extracted, and with the supporting the finding or text data explaining. Two researchers independently assessed whether the findings are consistent with the supporting data. Credibility assessment was provided for each finding: unequivocal, credible, or unsupported. The researchers studied the similarities and inconsistencies of coded text, and discussed to implement an intercoder agreement. A classification was created that can be used to represent the meaning of the initial data set. Finally, the researchers repeatedly evaluated these categories to determine similarities and summarize the synthesized results.

## Results

This analysis included nine studies from the following countries: China (*n* = 3), Turkey (*n* = 4), Spain (*n* = 1), and Korea (*n* = 1). These 9 studies involved 163 nursing students. All the included studies were descriptive qualitative analysis (*n* = 1), documentary analysis (*n* = 1), phenomenological approaches (*n* = 6), and descriptive exploratory design (*n* = 1). All studies published were original articles (Appendix Table 2). Three descriptive themes reflecting nursing students' experience in the ICU during their practicum: challenges of clinical practicum in the ICU, the expectation of support from multiple sources, and the importance and necessity of practicum in the ICU. The themes were divided into some subthemes of meaningful units, as shown in Appendix Table 3.

### Theme 1: Challenges of clinical practicum in ICU

#### Psychological change

Overall, this review found that every research reflected a various of psychological changes of nursing students early in their practicum (4, 11, 29–35), including, but not limited to, obscurity, fear, and anxiety. The nursing students stated that because many things in the ICU were different from the units they've seen before, they felt obscurity. “Most patients' eyes in intensive care were generally closed and most of them were intubated. Frankly, the patients here looked very different and scary” (4). Meanwhile, some students experienced fear because they felt incompetent and worried about doing something wrong and harming the patient in ICU during their first few days. “I helped the nurse to perform oral care. The patient was intubated. I was worried that I would hurt the patient, and the unconsciousness of the patient made me feel even more nervous” (33). Most nursing students admitted that they were anxious and nervous concerned about the ICU device and unconscious patients at the beginning of their clinical practicum. “When I first started my practicum in the ICU, I felt anxious and uneasy about the alarm sounds of various devices” (11).

#### Physical stress

The nursing work has a heavy workload because the diseases of ICU patients are complex and change rapidly. “Almost every day there are critically ill patients rescued, I feel tired from the heavy workload” (29). Nursing students found that not only was the illness of ICU patients stressful but the instruments and equipment used in treatment and care also increased their stress levels. “There were so many things I had to pay attention to in the ICU, IV fluids, the monitors, the patients…” (35). Although the number of patients in the ICU is small, they lack self-care. The patients are entirely dependent on nurses. “The nurse looked tired and exhausted” (33).

#### Challenges in caregiving

Nursing students stated that in the ICU practicum start stage, they were affected while aspirating secretions or providing tracheostomy and wound care for the ICU patients. In particular, they experienced nausea and disgust while performing care for some pressure sores. “Respiratory tract suction! The sound of suction bothers me, let alone doing it” (4). Further, we found that some students were challenged with caregiving due to limitations in knowledge and ability in the early days of practicum. “Intra-aortic balloon counter-pulsation is something that we have never heard in school, and we are very confused when we see it in the clinic” (31).

#### Challenges in communication

Patients in the ICU are in serious condition, and most of them are sedated and intubated and have life-threatening conditions. Nursing students not only feel a lack of knowledge but also feel inadequate in nursing communication. “I couldn't communicate with patients in the ICU” (33). Similarly, some students found that the ICU nurses also have difficulty communicating with the patients. “I felt that the nurse–patient interactions are ineffective even for the conscious patient, simply asking some questions cannot be regarded as communication” (33).

#### Challenges in ambivalent feelings related to death

Because of the severity and complexity of patients admitted to the ICU, students often have to face death. Death could be a source of huge stimulus, grief, and despair for nursing students who come face to face with it for the first time during their practicum (4). “It depresses me to know that a patient died there. But when I carefully, emotional thinking this question, I feel lonely and question myself” (4). However, some students criticized the team in charge for did not respond to the patient's death while others supported the team for not reacting.

#### Lack of belongingness

A sense of being accepted and recognized reported in nine researches was considered as an important factor in the process of learning. The lack of belongingness was one of the main challenges nursing students faced while doing a practicum in the ICU. A portion of the students felt alienation and loneliness in the ICU, perhaps due to the indifference of nurses and the environment of the ICU. “The environment was overwhelming for me” (33). The complicated patient needs, difficult treatments and procedures, and heavy workload in the ICU generally decreased the time spent by the nursing faculty members with the nursing students, inducing the latter's aloneness (36). “The nurses were so busy that they did not offer any help to the students” (32).

### Theme 2: The expectation of support from multiple sources

#### Expectation of support from the clinical instructors

The nursing students were keen to have their clinical instructors' support, which confirmed the importance of the instructors' encouragement in building students' confidence during the practicum. The positive attitude of clinical instructors had a positive impact on the overall learning process. “The clinical instructors were very friendly. They demonstrated and taught me how to provide patient care. That inspired me” (33). However, some nursing students often felt a lack of guidance from the instructors. “I hope the clinical instructors can pay attention to us” (31).

#### Expectation of support from curriculums

Due to numerous differences emerging between ICU and non-ICU clinical practicum, the participants realized that they lacked the appropriate specialized knowledge to implement their practicum skills, but indicated their desire to have the knowledge. “When we go into the ICU for the first time, if we were given some information about tools in intensive care, it would be great” (33).

### Theme 3: The importance and necessity of practicum in ICU

#### Gaining nursing competencies

Improvement in nursing students' competencies was an essential theme of the studies. Competencies were described as the complex student traits that entailed knowledge, skills, and attitudes (37). “I learned a lot in the ICU. I can apply what I have learned and improve my practical and theoretical knowledge” (4). Besides the enhancement of competencies, the nursing students also emphasized that their ability of multidisciplinary cooperation had also been greatly exercised, which subsequently helped them to complete various nursing tasks in a timely, accurate, and rapid manner. “Teamwork encouraged me during the practicum in ICU, we help each other with everything and no “so-and-so”s patient” (34).

#### Gradual adaptation and increased confidence

Some nursing students said that as the practicum progressed, they adapted to the ICU environment. “At first, I entered the ICU with uneasy. But I am getting used to intensive care every day” (4). As nursing students became more adaptable, they became more confident of their performance in the ICU. “After completing the internship in ICU, my strongest feeling was confidence. It's important to be able to follow up with patients and be alert enough. In this respect, I feel adequate.” (11).

#### Inspired professional values

Nursing students in this review who had both observed and done their practicum in the ICU realized that nurses played a key role in the ICU, which provided optimal, quality healthcare to intensive care patients as well as supported new nurses and students. “It would be impossible to manage the ICU without nurses. All responsibilities are given to the nurse, who plays an important role in intensive care” (4). As they became aware of the value of professionalism, the students also chose to become critical care nurses in the future. “I plan to work as a nurse in the ICU later, I understood what nursing is during my practicum in the ICU” (35).

## Discussion

After a manual search, nine qualitative studies on nursing students' experience in an ICU during their practicum were systematically reviewed on various databases and then integrated. The meta-synthesis results were considered to be scientific and reliable, and could complement qualitative research findings (38, 39). Compared with quantitative research, qualitative research can reflect real feelings and is widely applied in medical field, nursing, and health education (39). The main findings indicated that the nursing students faced abundant challenges while they were in the ICU during their practicum. Nursing students needed support from many sources to ensure the completion of the practicum. Finally, through a practicum in the ICU, the nursing students improved their competencies and knowledge. As their sense of professional values increased, their positive emotional experience and willingness to stay in the ICU also increased.

The review found that the most basic challenge for the nursing students when they first came to the ICU was the psychological change, including obscurity, fear, anxiety, and so on. Recent studies also highlighted that these key challenges were quite troublesome for nursing students during their practicum (40, 41). On the one hand, the nursing students felt inadequate and worried that they would harm the patient at the start of the clinical practicum because of the complexity of the ICU environment and the presence of critical patients (11). Some nursing students considered it as a state of feeling “powerless” (33). On the other hand, the nursing students were less experienced, which could have caused psychological change (4). A few students stated, however, that their psychological discomfort decreased with the support of the clinical instructors and the other members of the care team. Mutual support among members of the nursing team was important in adapting to the clinical process for nursing students (42).

The feeling of being part of a team created a positive atmosphere for effective learning, while the lack of belongingness was described by some nursing students as demotivating factor that hurt the learning process (32, 33). Therefore, it was necessary to develop a harmonious relationship between the clinical instructors and students to increase the sense of belongingness (43), which was a key to creating a positive learning environment (44). The clinical instructors were crucial components in establishing harmonious relationships. Previous research (45) showed that clinical instructors were simultaneously considered a potential source of stress and a major coping resource during practicum. For the most part, a consensus existed in the reviewed sources that students were satisfied with their relationships, interactions, and learning experience with the clinical instructors. However, some studies also indicated negative experiences (29, 31). On the one hand, the clinical instructors should establish their roles, not only to become clinical nurse role models but also to become knowledgeable, trustworthy teachers (46). On the other hand, hospital management departments should provide opportunities for clinical instructors to complete higher-level academic education and take psychology and pedagogy courses, which can facilitate them master flexible teaching ways and deepen their understanding of intensive care unit professional development. During the COVID-19 pandemic, critical care nurses assumed tremendous caring responsibility for critically ill patients at the same time. This required hospital management departments to formulate a standardized assessment mechanism for clinical instructors and to rationalize the allocation of ICU nursing human resources to prevent clinical instructors from burnout and exhaustion (46).

Another challenge for nursing students emphasized in our review was the physical stress they encountered while doing a practicum in the ICU. Perhaps the nursing students were in the ICU for the first time. They had no specialized knowledge of intensive care nursing, and the clinical instructors could not cooperate due to their heavy workload. Hence, strategies were needed to enhance students' specialized knowledge in the ICU. A previous study found the modules education very useful for nursing student learning and rated their confidence and knowledge levels as high (47–49). Module course content included strengthening students' specialized knowledge and health assessment of the cardiovascular, respiratory, and gastrointestinal systems, guided reflection, clinical case studies, and instruction. Besides, a few nursing students' recommendations for the ICU curriculum included an emphasis on the explanation of technical equipment and equipment used in the ICU (11).

Nursing students also faced important challenges in caregiving due to the differences between theoretical knowledge and practice (4, 29, 31). It was also anticipated that some nursing students would affect caregiving due to a lack of specialized experience while giving interventions such as aspiration and wound and tracheostomy care. Also, many nursing students noted differences in the expectations of college educators, clinical instructors, and nursing faculty members concerning clinical course objectives and skills performance (50). We suggested some measures that could help bridge the theory–practice gap. First, close cooperation should exist between college educators, clinical instructors, and hospital management for effective clinical practicum education. The nursing curriculum should essentially be aligned with the clinical environment to ensure that nursing students are ready for the challenges of the dynamic complex medical system while doing a practicum in ICU. Also, planners of nursing education should devote more energy to solving this problem. Second, previous studies (47, 51–53) noted that the clinical simulations were effective and useful for nursing students for their preparedness and practicum in the ICU. All of the simulations had high fidelity and included audio–video recordings of ICUs, clinical scenarios, and mannequins of deteriorating patients (54). Critical illness simulations included acute upper gastrointestinal hemorrhage, hypovolemic and septic shock, sudden death, multiple injuries, acute myocardial infarction, and so on. Advanced oxygen delivery, hemodynamic monitoring, communication with intensive care teams, critical thinking, clinical reasoning, and reflection were mastered by nursing students through simulated clinical scenarios. The clinical simulation could also improve nursing students' sense of self-efficacy, which had a positive main effect on staying effective and positive during clinical practicum (55–57). Finally, hospital managers could adopt the experiential learning method based on the Kolb model (58). Some researchers (59) found that the self-learning ability and ICU clinical competencies of nursing students could be improved through the experiential learning method with four stages: real experience, observation and reflection, abstract concept, and active verification.

One of the purposes of nursing clinical practicum education was that nursing students should improve communication skills while learning clinical nursing knowledge. The ability to communicate effectively will promote the development of professional identity (60). A few nursing students' recommendations for ICU clinical practicum included developing nursing students' ability to communicate with patients in serious conditions (11). Some feelings and emotions, such as pain, sadness, discomfort, and fear, could be enhanced and better expressed through non-verbal cues such as eye contact, gestures, and expression. A previous study (61) proved that clinical simulation should be used as a good learning strategy to educate and train nursing students in communication skills. Besides, the clinical instructors and college educators had important responsibilities in solving nursing student communication problems with intensive care patients, which could be improved by modeling good practices and having clinical instructors and clinical nurses as role models and cases.

Another important finding was that nursing students gained access to care for terminal patients when they practiced in the ICU. Death was a life experience that nursing students encountered rarely in a non-ICU setting (62). The experience affected their painful feelings and questioned whether the patient's death was caused by any inadequacy in treatment or care. An increase in clinical end-of-life content in nursing education programs and the need for debriefing sessions for all students caring for the dying are essential (63). Noteworthy, the focus in our curriculum was on the patient and his/her family during end-of-life care (11). However, how nurses coped with this was missing. College educators and clinical instructors should help students turn this sad experience into a positive learning experience (62). For example, nursing students learn about nursing and communication with a terminal patient with the help of clinical instructors. Also, during this stage, how to break the news of a patient's death to the patient's family is critical (23). Another research (33) showed that clinical instructors should accompany the students to overcome painful emotions when facing a patient's death, which illustrated the educational significance of the process for students as well as the significance of the experience.

Consistent with previous study results (11), a review found that most of the nursing students in the ICU had learned “very much” compared with non-ICU students. First, practicum in the ICU is crucial, allowing them to identify and master the care of patients with difficult and complicated diseases, improve their professional skills for working in interdisciplinary environments, and develop critical thinking and communication ability. Second, nursing students have the opportunity to perform technical procedures (e.g., airway management, vascular access placement) to address the basic needs of patients (e.g., physical comfort, end-of-life care), which were difficult to obtain in other departments. Moreover, nursing students in the ICU might also develop emergency skills, such as quickly recognizing deteriorating patients and administering cardiopulmonary resuscitation. The complicated patient situation and complex structure of the ICU environment, which initially caused their uneasy, enabled them to learn a lot as their practicum progressed and as they came to adapt to the environment (4). Additionally, some nursing students had increased self-confidence after completing their practicum in the ICU and wanted to be intensive care nurses following graduation. As mentioned in the review, practicum in the ICU was expected to help nursing students consolidate the competencies they had gained and learn how to care for patients with complex needs and improve their self-confidence and professional values.

## Conclusion

Psychological change, physical stress, challenges in caregiving, challenges in communication, challenges in ambivalent feelings related to death, and lack of belongingness were key factors that impacted nursing students' learning during their practicum in the ICU. Understanding nursing students' expectations is important to take appropriate measures to overcome challenges. The findings of this review suggested that nursing students considered doing one's practicum in the ICU as a positive experience despite the challenges involved. Thus, appropriate guidance and monitoring should be provided to the nursing students in the ICU, such as the simulation education of ICU nursing, modules education, experiential learning method, death education, communication training, competent clinical instructors, and so on. This research also had implications for colleges and clinical institutions by promoting opportunities for collaboration and developing policies with a focus on reducing the potential gaps between theory and clinical practicum. It was speculated that these results would help to further research, to be better prepared and improve the quality of ICU nursing education when responding to ICU care needs in the future.

## Limitations

There were several limitations in this meta-synthesis. According to the inclusion and exclusion criteria, only qualitative researches in Chinese or English published in indexed journals were included. Researchers did not search dissertations and gray literature, which might cause an information bias. Nursing education for ICU practicum in different regions might result in various protocols and policies that might affect nursing students' perception and experience in the ICU. Finally, this review represents the authors with varied perspectives, which may have diverse results.

## Data availability statement

The original contributions presented in the study are included in the article/Supplementary material, further inquiries can be directed to the corresponding authors.

## Author contributions

YL: conceptualization, methodology, formal analysis, writing—original draft, and writing—review and editing. LW: conceptualization, methodology, writing—original draft, and writing—review and editing. HS and PH: conceptualization, methodology, formal analysis, and writing—review and editing. JJ and XD: methodology, formal analysis, and writing—review and editing. All authors contributed to the article and approved the submitted version.

## Conflict of interest

The authors declare that the research was conducted in the absence of any commercial or financial relationships that could be construed as a potential conflict of interest.

## Publisher's note

All claims expressed in this article are solely those of the authors and do not necessarily represent those of their affiliated organizations, or those of the publisher, the editors and the reviewers. Any product that may be evaluated in this article, or claim that may be made by its manufacturer, is not guaranteed or endorsed by the publisher.
