# Supplementary material for: Nursing students' experience during their practicum in an intensive care unit: A qualitative meta-synthesis
Source: Front Public Health. 2022 Sep 29;10:974244. doi: 10.3389/fpubh.2022.974244 (PMC9556851; doi:10.3389/fpubh.2022.974244)
Supplement: Supplementary file 1 [file Data_Sheet_1.docx]

**APPENDIX:**

Records after duplicates were removed (*n* = 208)

Full-text studies assessed for eligibility (*n* = 9)

Literature excluded:

irrelevant to the subject, unable to obtain the full text, not in English or Chinese (*n* = 183)

Qualitative studies included (*n* = 9)

Literature excluded:

incomplete information, focused on nurses who were not in direct care or care for noncritical patients, not an original study (*n* = 16)

Records identified by tracing references (*n* = 2)

Records identified through database searching (*n* = 376):

Cochrane Library (*n* = 47), PubMed (*n* = 43), Embase (*n* = 67), *Ovid* (*n* = 12), Elsevier (*n* = 3),Web of Science (*n* = 132), CBM (*n* = 20) , CECDB (*n* = 22),CNKI (*n* = 20) and VIP (*n* = 10)

Records screened (title + abstract) (*n* = 25)

Quality assessment of included studies (*n* = 9)

Figure 1. PRISMA flowchart.

Table 1. Results of quality assessment based on the criteria of the Joanna Briggs Critical Assessment Tool for Methodological Quality Assessment

| Study | Q 1 | 2 | 3 | 4 | 5 | 6 | 7 | 8 | 9 | 10 | Result (%) |
| --- | --- | --- | --- | --- | --- | --- | --- | --- | --- | --- | --- |
| Jiang et al. (2017) | Y | Y | Y | Y | Y | Y | Y | Y | Y | Y | 20/20 (100%) |
| Xia et al. (2016) | Y | Y | Y | Y | Y | N | N | Y | U | Y | 15/20 (75%) |
| Yu et al. (2020) | Y | Y | Y | Y | Y | N | N | Y | U | Y | 15/20 (75%) |
| Sim et al. (2021) | Y | Y | Y | Y | Y | U | Y | Y | Y | Y | 19/20 (95%) |
| Saglam et al. (2020) | Y | Y | Y | Y | Y | Y | N | Y | Y | Y | 18/20 (90%) |
| Yüksel et al. (2020) | Y | Y | Y | Y | Y | N | U | Y | Y | Y | 17/20 (85%) |
| Gonzalez-Garcia et al. (2020) | Y | Y | Y | Y | Y | Y | U | Y | U | Y | 18/20 (90%) |
| Vatansever et al. (2016) | Y | Y | Y | Y | Y | N | N | Y | Y | Y | 16/20 (80%) |
| Tastan et al. (2015) | Y | Y | Y | Y | Y | Y | N | Y | Y | Y | 18/20 (90%) |

Critical appraisal (*n* = 10) (Y = yes; N = no; U = unclear).

Q: question.

Q1= Is there congruity between the stated philosophical perspective and the research methodology? Q2=Is there congruity between the research methodology and the research question or objectives? Q3=Is there congruity between the research methodology and the methods used to collect the data? Q4=Is there congruity between the research methodology and the representation and analysis of data? Q5=Is there congruity between the research methodology and the interpretation of results? Q6=Is there a statement locating the researcher culturally or theoretically? Q7=Is the influence of the researcher on the research and vice versa addressed? Q8=Are participants and their voices adequately represented? Q9=Is the research ethics according to the current criteria or for recent studies, and is there evidence of ethics approval by an appropriate body? Q10=Do the conclusions drawn in the research report flow from the analysis or interpretation of the data?

Table 2. Description of the included studies

| *Author (year)/country* | *Research method* | *Participants* | *Aims* | *Results* |
| --- | --- | --- | --- | --- |
| Jiang et al. (2017)/China | Phenomenological research; semistructured interviews | Seventeen nursing students who had clinical practicum in ICU from a hospital in Shanghai | Explore the stress experience and coping style of practicum nursing  students in ICU | Four themes: troubled by a lack of knowledge, strong physical stress, negative emotional stress, and multiple coping styles |
| Xia et al. (2016)/China | Phenomenological research; semistructured interviews | Twelve undergraduate nursing students who had clinical practicum in PICU from a hospital in Fujian province | Understand the expectations and practicum experience of the undergraduate nursing students in the process of practicum in the PICU, and provide a reference for making more rigorous clinical teaching plans for the future and nursing teaching | Seven themes in 2 stages: pre-internship themes were stress, anxiety, and expectation to achieve self-worth; post-internship themes were higher anxiety, increased empathy, low self-confidence, and limited self-actualization |
| Yu et al. (2020)/China | Phenomenological research; semistructured interviews | Twelve nursing students who had clinical practicum in CCU from a hospital in Beijing | Understand the sources and responses to the negative emotions in nursing students during their CCU internship and improve the nursing clinical teaching | Eight themes: insufficient psychological adjustment ability, lack of patients' trust, basic nursing workload, high tension in the work environment, and lack of specialized knowledge in the face of these negative emotions. Most nursing students used a variety of ways to actively deal with negative emotions and desired to get attention from others |
| Sim et al. (2021)/Korean | Phenomenological approach; semistructured interviews | Fifteen nursing students who had clinical practicum in the newborn nursery and neonatal intensive care unit from a hospital in Seoul | Understand the clinical experience of nursing students in newborn nurseries and neonatal intensive care units and provide basic data for developing strategies to promote effective clinical education | Four themes: expectations and anxieties about clinical practicum, gaining extensive knowledge about neonatal nursing, challenges faced in clinical practicum, and experiencing changes in interpersonal relationships |
| Saglam et al. (2020)/Turkey | Phenomenological approach; semistructured interviews | Twenty-five nursing students who had clinical practicum in the two different ICUs from a university hospital in Turkey | Describe the nursing student's ICU internship experience | Seven themes: fear, awareness of the nurse's role, finding opportunities for self-improvement, difficulties in nursing, difficulties in communicating with patients and the nursing team, experiencing conflicting emotions related to death, and adjusting to the clinical environment |
| Yüksel et al. (2020)/ Turkey | Phenomenological approach; semistructured interviews | Twenty-one second-year nursing students who received clinical training as a part of the Surgical Diseases Nursing course in the neurosurgical ICU of a university hospital | Explore the nursing students' perspectives, thoughts, and clinical experience of neurosurgical ICU practicum | Five themes: perceptions (features of the environment, patients’ condition and nurses’ characteristics) emotional effects (patients’ condition care interventions) communication (student–patient communication, nurse–patient communication, and student–nurse communication) contribution to clinical training (putting theoretical knowledge into practice and professional gain) opinions |
| Gonzalez-Garcia et al. (2020)/Spain | Qualitative methodology; a documentary analysis | Twenty-eight reflective learning journals were carried out at a public university in Northern Spain | Explore the experience of final year nursing students during their clinical practicum in the emergency department and ICU | Five themes: a strong emotional experience, the importance of attitudes over technology, identifying with nurses who dominate their environment and are close to the patient in complex and dehumanized units, how to improve care in critically ill patients, and how to support their families, the experience of these clinical rotations is different between girls and boys. |
| Vatansever et al. (2016)/Turkey | Descriptive exploratory design; semistructured interviews | Eighteen nursing students who had clinical practicum in the surgical ICUs from a university hospital in Turkey | Explore the impact of ICU experience on nursing students' learning | Four themes: perspectives on the ICU environment and patients, perceptions of becoming an ICU nurse, understanding of ICU patient communication and empathy, and the contribution of patient care activities in study |
| Tastan et al. (2015)/ Turkey | Descriptive qualitative approach; open-ended and in-depth audio-taped interviews | Fifteen 4th-year baccalaureate nursing students who had clinical practicum in the ICUs from a military medical academy in Turkey | Describe the clinical experience of undergraduate nursing students in the ICU | Ten themes: anxiety, fear of causing harm, emotional connection and empathy, developing self-confidence, developing a sense of responsibility for the patient, prioritizing patient care, maintaining dignity, to face the situation, communication in the ICU, and curriculum needs |

Note: PICU= Pediatric Intensive Care Unit; CCU=Coronary Care Unit

Table 3. Thematic synthesis findings

| *Descriptive themes:* | *Subthemes:* |
| --- | --- |
| **Challenges of clinical practicum in ICU** | Psychological change |
|  | Physical stress |
|  | Challenges in caregiving |
|  | Challenges in communication |
|  | Challenges in ambivalent feelings related to death |
|  | Lack of belongingness |

| **Expectation of support from multiple sources** | Expectation of support from the clinical instructors |
| --- | --- |
|  | Expectation of support from the curriculums |
| **Importance and necessity of practicum in ICU** | Gaining nursing competencies |
|  | Gradual adaptation and increased confidence |
|  | Inspired professional values |
